# A case-control study of sporadic Creutzfeldt-Jakob disease in Switzerland: analysis of potential risk factors with regard to an increased CJD incidence in the years 2001–2004

**DOI:** 10.1186/1471-2458-9-18

**Published:** 2009-01-14

**Authors:** Jessica Ruegger, Katharina Stoeck, Lorenz Amsler, Thomas Blaettler, Marcel Zwahlen, Adriano Aguzzi, Markus Glatzel, Klaus Hess, Tobias Eckert

**Affiliations:** 1 Department of Neurology, University Hospital Zurich, Zurich, Switzerland; 2 Institute of Neuropathology, University Hospital Zurich, Zurich, Switzerland; 3 Department of Neurology, University Hospital Hamburg-Eppendorf, Hamburg-Eppendorf, Hamburg, Germany; 4 Federal Office of Public Health, Bern, Switzerland; 5 CSL Behring, Bern, Switzerland; 6 Bristol-Myers Squibb, Wallingford, CT, USA; 7 Institute of Social and Preventive Medicine, University of Bern, Bern, Switzerland; 8 Institute of Neuropathology, University Hospital Hamburg-Eppendorf, Hamburg-Eppendorf, Hamburg, Germany; 9 Swiss Tropical Institute, Basel, Switzerland

## Abstract

**Background:**

In 2001, the observed annual mortality from Creutzfeldt-Jakob disease (CJD) in Switzerland increased from less than 1.5 to 2.6 per million inhabitants. An underlying cause could not be identified.

**Methods:**

To analyse potential risk factors for sCJD in Switzerland, close relatives of 69 sCJD-patients and 224 frequency age-matched controls were interviewed in a case-control study using a standardised questionnaire. 135 potential risk factors including socio-demographics, medical history, occupation and diet were analysed by logistic regression adjusting for age, sex and education.

**Results:**

sCJD patients were more likely to have travelled abroad, worked at an animal laboratory, undergone invasive dental treatment, orthopaedic surgery, ophthalmologic surgery after 1980, regular GP visits, taken medication regularly, and consumed kidney. No differences between patients and controls were found for residency, family history, and exposure to environmental and other dietary factors.

**Conclusion:**

Although some factors were significantly more frequent among sCJD-cases, this study did not reveal specific explanations for the increased incidence of deaths due to sporadic CJD observed in Switzerland since 2001. Results have to be interpreted with caution due to multiple testing and possible recall bias in association with a long incubation period. The most plausible reason for the increase in Swiss sCJD cases after 2000 is an improved case ascertainment. Therefore, underreporting of cases might well have occurred before the year 2001, and the "real" yearly incidence of sCJD might not be lower than, but rather above 2 per million inhabitants.

## Background

Creutzfeldt-Jakob disease (CJD) is a rare but inevitably fatal neurological disorder. CJD is associated with spongiform changes in the brain and an accumulation of a misfolded protein termed prion, which is believed to be the agent responsible for transmission of the disease.[1] In most industrialised countries, CJD is recorded with a yearly incidence of 1.0–1.5 cases per million inhabitants.[2] There are several pathogenically distinct forms of CJD, of which the classical forms (sporadic, genetic and iatrogenic) have been known for decades. Another form, variant CJD (vCJD), was first described in the United Kingdom in 1996, and consensus exists that vCJD is caused by ingestion of bovine products, particularly of so-called "risk material" from cattle infected with Bovine spongiform encephalopathy (BSE).[3,4] Among the three classical forms, genetic CJD (gCJD) develops due to specific mutations in the prion gene, while iatrogenic CJD (iCJD) has resulted from contaminated blood, human pituary gland hormone products, dura mater and corneal grafts, and during neurosurgery via surgical instruments and stereotactic EEG electrodes from one patient to another. [5-11] However, after the implementation of preventive measures such as more efficient sterilisation procedures and prohibiting the use of human dura mater, iatrogenic cases were observed less frequently.[12] Sporadic CJD (sCJD) is by far the most common of all CJD forms, accounting for 80 to 90% of all cases.[13] Its aetiology remains unknown.[14]

**Figure 1:**
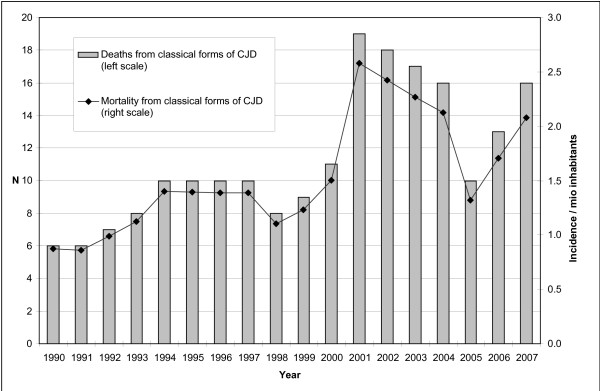
**Reported deaths from "classical forms" of definite and probable CJD (i.e. sporadic, familial and iatrogenic) in Switzerland (1990–2007)**.

In Switzerland, mandatory reporting of definite CJD cases was introduced in 1987, and since 1999 all clinically suspected CJD cases must be reported as well. In the year 2001, the annual mortality rate increased significantly to 2.6 per million inhabitants from around 1.4 per million observed during the 1990ies.[15] Figure 1 shows yearly incidences of reported CJD deaths in Switzerland: between 1988 and 2000, an average of 9 definite and probable CJD deaths according to European criteria (maximum: 11 cases in 2000) were reported annually.[16] In 2001, 19 deaths were reported, an increasing trend that was sustained in the following years with 18 deaths in 2002, 17 in 2003, 16 in 2004, 10 in 2005, 13 in 2006 and 15 deaths in 2007. This increase in reported CJD-deaths was observed in all parts of the country and without any indications for a geographical change. All deaths in the period of 2001–2004 were due to classical forms of CJD, and until November 2008, no case of vCJD has been recorded in Switzerland.[17]

A comparable increase in CJD-cases has not been observed in Switzerland's neighbouring countries (Austria, Germany, France and Italy). Corresponding figures are available on the website of The European and Allied Countries Collaborative Study Group of CJD (EUROCJD).[16] The reason for this sudden rise in the annual death rate for CJD in 2001 in Switzerland was unclear, and was therefore investigated in a case-control study. Three main hypotheses have been discussed: the observed increase could have been caused by a) a zoonotic cause similar to the transmission of BSE in patients with vCJD, b) an iatrogenic cause or c) ascertainment bias due to a heightened perception and awareness level of the disease in physicians.[18,19] The two former hypotheses would refer to a so far unknown route of transmission for sporadic CJD, since the sCJD cases included in this study did not show any of the characteristic pathological findings of vCJD. Furthermore, they have not been exposed to any of the known iatrogenic risk factors like cadaveric derived pituary hormones or dura mater grafts. Blood transfusion has been shown to be a potential route of vCJD transmission and could therefore be a potential risk factor for other forms of CJD.[11,20] As far as the BSE epidemic in cattle is concerned, Switzerland was among the countries in Europe experiencing an early and high rise in BSE cases, even if the incidence remained significantly below that observed in the United Kingdom.[21] Therefore, with rising case numbers of vCJD in the United Kingdom, the question arose if the "surplus" of sporadic cases in Switzerland could be related to dietary risk factors in analogy to the development of vCJD.

The principal aim of this study was to investigate whether there are specific exposures that could explain the rise in mortality rates for sCJD observed in Switzerland in the years 2001–2004. A focus was set on external and thus preventable risk factors.

## Methods

### Case-patients

All cases included in this study were sCJD patients with residence in Switzerland recorded by the mandatory Swiss infectious disease notification system between January 2001 and December 2004 (n = 72). Surveillance of CJD is carried out by the Swiss Federal Office of Public Health (SFOP) in collaboration with neurologists, neuropathologists, psychiatrists, cantonal physicians and the National Reference Centre for Prion Diseases (NRPE) in Zürich. During the recruitment time 2001–2004, two genetic CJD cases as well as one iatrogenic case (due to a dura mater graft) occurred, which were not included in this study. In addition, cases were excluded if their relatives and/or physicians did not consent to the study (n = 3), resulting in 69 patients with sCJD included in the study. Among these 69 cases, diagnosis of CJD was histologically and biochemically confirmed in 61 patients (88.4%). Eight patients (11.6%) were classified as probable sCJD cases according to the criteria of the European Creutzfeldt-Jakob Disease Surveillance Network EuroCJD.[16] Codon 129 M/V polymorphism was analysed in all CJD patients where blood or frozen brain samples were available. PRNP sequencing was performed in samples where written consent was given by the patient or close relatives. A description of the analysis of the genetic markers was published elsewhere.[22] The interviewed proxy for patients was either the spouse, a close relative or in a few instances another close person such as a friend, who had known the patient for several years.

### Controls

Data of 224 controls were included in this study. To limit potential selection bias, controls were recruited in two different ways. The first group (n = 69) was recruited from general practitioner (GP) offices between November 2004 and July 2005. The second group (n = 155) was recruited by random digit telephone dialling from June to September 2005.

Controls recruited from GP offices were matched by age and district of residence, and were chosen randomly by the following procedure: the GP was asked to select from his collection of patient records in an alphabetic order the next suitable matching control record following his own family name. This record was excluded if the age was more than 5 years different from that of the corresponding control patient. Potential control subjects were excluded from participation if they were suffering from a severe disease or were known to their GP as being a very non-compliant patient. For each GP, a maximum of three corresponding controls were included. A standard model letter with an *informed consent *enclosed was sent to the matched control and a face-to-face interview was arranged.

In order to compare cases with the general population, the second control group (n = 155) was recruited by random digit telephone dialling based on the Swiss Telephone registry [Twixtel Version 32 (5/2005)]. These control subjects were frequency matched by 5-year age group, sex and residency within the same language region of Switzerland. According to the Swiss Federal Statistical Office (FSO), 71% of the Swiss population was reachable by a fixed line telephone number in December 2004.

### Data collection

For both, cases and controls, a standardised questionnaire was used, which was translated from English into German, French and Italian. This questionnaire had previously been used in case-control studies in the United Kingdom and other countries participating in EUROCJD network.[23,24] A total of 135 different items on social factors, residential history, travel history, family history, previous medical history (surgery, medical treatments and drug use), as well as potential environmental (occupation, animals and farming) and dietary risk factors were analysed. As an example for previous medical history, controls and proxy persons for the CJD-patient were asked if they had ever taken any medications regularly (i.e. for a period > 1 month) since the beginning of 1980. Respondents were told not to include treatments related to CJD or their current disease, respectively.

### Ethical issues

Data collection of cases is an integral part of the intensified CJD surveillance in Switzerland which became effective in November 2002 by a decree of the Federal Council. The database is registered at the Swiss Federal Data Protection Commissioner, and the study was approved by the ethics committee of the Canton of Zurich. All data were analysed in an anonymous manner.

### Statistical methods and analysis

Power calculations for an unmatched case-control study were performed prior to collecting data on control subjects. The calculations were performed for the total of both control groups. For 69 cases and 224 controls, assuming a prevalence for a certain exposure of 25.0% in cases and of 10.0% in controls (resulting in an odds ratio of 2.96), the study exhibited a power of 80% at the α = 5% confidence level. Cases were not only compared with all controls compiled, but also with each of the two control groups in separate subgroup analyses. The direct matching in the GP control group, e.g. controls having the same GP as the cases, was only possible for one third of all patients. Therefore the data for both control groups was analysed as an unmatched case-control study.

All potential risk factors were analysed by summary statistics, logistic regression. The latter included univariable models (= crude estimates) and models including age, sex and number of school years (= adjusted estimates). "Number of school years" was chosen as a proxy parameter for education and socio-economic status. Age, "number of school years" and the "number of surgical operations" were taken as continuous variables. All other variables – including frequency of food consumption – were taken as a binary variable into the models. Main outcome measures were odds ratios for assessed exposures with their corresponding 95% confidence intervals and two-sided p-values. P-values less than 0.05 were considered significant. As computer program for data collection and graphics we used Microsoft "Excel 2000". For power calculations we used "Statcalc" in EpiInfo Version 6. For data analysis we used "Intercooled STATA" version 8.0.[25]

## Results

### Recruitment of controls

#### GP controls

69 general practitioner control subjects were recruited. For 19 of the CJD patients, three corresponding controls were interviewed, and for another six patients two corresponding controls were interviewed. None of the addressed control persons declined participation. However, the GP's of the remaining 44 patients rejected to participate in the study, and for these patients, no direct control subjects were interviewed.

#### Telephone controls

2272 telephone numbers were chosen by random digit dialling, and a letter informing about the study beforehand was sent to the corresponding addresses. In households with valid telephone numbers (n = 2095), the response rate was 68%, which corresponds to 1422 screened households. In 1035 of these households, no target person of matching age, sex or residence requirements could be identified. Among 387 identified target persons, 156 interviews were completed, of which one could not be used due to missing data. 231 target persons declined participation. Thus the final response rate of target persons was 40%.

### Associations of sporadic CJD with potential risk factors

#### Socio-demographic, residential and other background factors

Associations of sCJD with age, sex, country of birth, education, marital status, and residence in German versus French and Italian speaking parts of Switzerland are shown in table 1. Other potential risk factors such as left versus right handedness, family history of dementia and travel history are also shown in this table. When adjusted for age, sex and education, sCJD patients more often travelled abroad (OR 9.31 95% CI (3.63–23.91). Controls on the other hand had travelled more often to tropical destinations (OR 0.43, 95% CI 0.19–1.00) and to the U.K. (OR 0.29, 95% CI 0.10–0.86), the country experiencing the highest BSE-incidence after 1980. Also, control subjects had completed more years of education (OR 0.88, 95% CI 0.80–0.97) than sCJD patients. No differences were found with respect to age, sex, place of birth and living or a family history of dementia.

**Table 1:** Sociodemographic factors

				**comparison with all control subjects**					
**risk factor**	**n** **total**	**mean (SD) or %**		**crude estimate**		**age and sex adjusted estimate**		**age, sex and education adjusted estimate**	
		**cases** **n = 69**	**controls n = 224**	**OR (95% CI)**	**p**	**OR (95% CI)**	**p**	**OR (95% CI)**	**p**
age (years)	293	68.7 (9.6)	69.6 (8.2)	0.99 (0.96–1.02)	0.449	0.99 (0.96–1.02)^1^	0.444	0.99 (0.96–1.02)	0.450
age-group 41 – 59 years	35	15.9	10.7	1^5^		1^5^		1^5^	
age-group 60 – 69 years	103	33.3	35.7	0.63 (0.27–1.47)	0.283	0.63 (0.27–1.47)	0.281	0.61 (0.25–1.50)	0.282
age-group 70 – 79 years	112	36.2	38.8	0.63 (0.27–1.45)	0.277	0.61 (0.26–1.42)	0.249	0.61 (0.25–1.49)	0.278
age-group 80 – 85 years	43	14.5	14.7	0.66 (0.24–1.81)	0.420	0.68 (0.25–1.86)	0.452	0.65 (0.23–1.87)	0.427
sex (male)	293	65.2	57.1	1.41 (0.81–2.47)	0.234	1.41 (0.80–2.47)^1^	0.232	1.70 (0.93–3.08)	0.080
education: ≥ 10 school years	289	44.6	71.4	0.32 (0.18–0.57)	0.000	0.29 (0.16–0.52)	0.000	0.29 (0.16–0.52)	0.000
education: N° of school years	289	11.0 (3.6)	12.1 (3.2)	0.90 (0.82–0.98)	0.021	0.88 (0.80–0.97)	0.009	0.88 (0.80–0.97)	0.009
education: ≤ 9 school years grouped	100	55.4	28.6	1^5^		1^5^		1^5^	
education: 10–12 school years grouped	79	18.5	29.9	0.32 (0.15–0.67)	0.002	0.31 (0.15–0.66)	0.002	0.31 (0.15–0.66)	0.002
education: ≥ 13 school years grouped	100	26.2	41.5	0.32 (0.17–0.63)	0.001	0.31 (0.16–0.61)	0.001	0.28 (0.14–0.55)	0.000
marital status (married)	293	79.7	72.8	1.47 (0.76–2.83)	0.250	1.32 (0.67–2.61)	0.425	1.32 (0.65–2.67)	0.445
having a partner	293	79.7	73.7	1.40 (0.73–2.71)	0.311	1.25 (0.63–2.28)	0.527	1.24 (0.61–2.53)	0.551
residence in German speaking cantons (vs. French & Italian speaking)	293	76.8	75.9	1.05 (0.56–1.99)	0.876	1.08 (0.56–2.07)	0.822	1.03 (0.52–2.02)	0.931
country of birth (foreign^2^)	292	13.2	19.2	0.64 (0.30–1.40)	0.263	0.64 (0.29–1.38)	0.254	0.69 (0.31–1.52)	0.355
having lived abroad^2 ^(> 1980)	289	6.1	4.0	1.53 (0.46–5.15)	0.489	1.46 (0.42–5.04)	0.549	1.60 (0.46–5.74)	0.471
frequent travels abroad (= once per year, > 1980)^4^	119	83.3	35.4	9.13 (3.80–21.97)	0.000	8.61 (3.47–21.34)	0.000	9.31 (3.63–23.91)	0.000
travel history to the U.K. (> 1980)	276	7.6	22.4	0.28 (0.10–0.82)	0.020	0.27 (0.09–0.79)	0.017	0.29 (0.10–0.86)	0.026
travel history to tropical countries (> 1980)	276	15.1	28.3	0.45 (0.20–1.01)	0.053	0.44 (0.19–0.99)	0.046	0.43 (0.19–1.00)	0.045
left-handed^3 ^(vs. right)	290	9.1	10.7	0.83 (0.33–2.13)	0.704	0.83 (0.32–2.14)	0.701	0.76 (0.29–1.98)	0.577
dementia in family history	291	19.4	15.6	0.77 (0.38–1.56)	0.466	0.78 (0.38–1.59)	0.494	0.64 (0.30–1.37)	0.252

1) only sex or age adjusted, respectively 2) other than Switzerland 3) including ambidextrous 4) GP controls only 5) reference category

#### Environmental factors

Results of cases and controls with respect to occupation, farming related exposure and pet animals are shown in table 2. Cases had worked significantly more often in an animal laboratory (OR 9.6; 95% CI 1.4–64.5). Controls more often owned a pet rodent (OR 0.32, 95% CI 0.16–0.66). No differences between sCJD patients and controls were found with respect to all other work-related factors, such as occupations in the medical care setting, animal farming or the meat industry. Likewise there were no differences in exposure to leather products, fertilisers containing hoof and horn material, and with respect to farm-stays.

**Table 2:** Environmental risk factors – occupational exposure, farming related exposure and pets (> 1980)

				**comparison with all control subjects**					
**risk factor**	**n** **total**	**mean (SD) or %**		**crude estimate**		**age and sex adjusted estimate**		**age, sex and education adjusted estimate**	
		**cases n = 69**	**controls n = 224**	**OR (95% CI)**	**p**	**OR (95% CI)**	**p**	**OR (95% CI)**	**p**
medical profession	290	6.0	6.3	0.95 (0.30–2.98)	0.927	1.21 (0.36–4.09)	0.764	1.49 (0.43–5.15)	0.533
animal laboratory	290	4.5	0.9	5.18 (0.82–31.65)	0.075	5.32 (0.86–33.00)	0.073	9.55 (1.41–64.49)	0.021
pharmaceutical laboratory	290	1.5	0.5	3.36 (0.21–54.51)	0.393	3.45 (0.21–56.29)	0.385	3.39 (0.21–55.20)	0.390
research laboratory	290	1.5	2.2	0.66 (0.08–5.75)	0.707	0.62 (0.07–5.40)	0.662	0.68 (0.08–5.99)	0.730
work with animals (animal care, veterinarian)	290	14.9	8.1	2.00 (0.87–4.57)	0.101	2.06 (0.89–4.80)	0.092	1.45 (0.59–3.55)	0.419
work in meat industry	290	3.0	3.6	0.83 (0.17–4.00)	0.813	0.83 (0.17–4.05)	0.816	0.67 (0.13–3.33)	0.622
work in catering industry	290	6.0	2.2	2.77 (0.72–10.61)	0.138	3.07 (0.78–12.05)	0.108	2.81 (0.70–11.27)	0.144
work with animal products (e.g. leather)	290	4.5	3.1	1.44 (0.36–5.75)	0.600	1.43 (0.36–5.74)	0.616	1.45 (0.35–5.96)	0.610
farm work or stay (for > 1 week)	289	10.6	14.4	0.71 (0.30–1.69)	0.436	0.70 (0.29–1.68)	0.431	0.62 (0.25–1.50)	0.287
work with hoof or horn	288	15.4	13.0	1.22 (0.56–2.65)	0.622	1.23 (0.56–2.69)	0.604	1.12 (0.49–2.53)	0.794
work with bonemeal	287	6.3	8.5	0.72 (0.23–2.18)	0.557	0.67 (0.22–2.06)	0.484	0.75 (0.24–2.33)	0.617
work with dried blood	289	3.0	3.6	0.84 (0.17–4.05)	0.828	0.79 (0.16–3.85)	0.770	0.75 (0.15–3.83)	0.733
work with fertiliser	289	24.2	22.0	1.14 (0.60–2.17)	0.698	1.15 (0.60–2.20)	0.677	1.14 (0.59–2.22)	0.692
pet cat	289	31.8	46.2	0.54 (0.30–0.97)	0.040	0.55 (0.31–0.99)	0.046	0.57 (0.32–1.04)	0.067
pet dog	289	39.4	30.5	1.48 (0.84–2.62)	0.177	1.48 (0.84–2.62)	0.179	1.52 (0.85–2.72)	0.161
pet rodent	288	16.9	36.8	0.35 (0.17–0.71)	0.003	0.32 (0.16–0.66)	0.002	0.32 (0.16–0.66)	0.002

#### Previous medical history

Exposure of cases and controls to surgery is shown in table 3. Results for other invasive medical procedures such as lumbar punctures, injections, blood donation, transfusions, regular medication as well as non-invasive medical risk factors such as allergies, cigarette smoking and illegal drugs are listed in table 4. sCJD patients significantly more often had undergone orthopaedic surgery (OR: 4.77; 95% CI: 2.60–8.83), even if limiting the analysis to those who had been operated after 1980 (OR: 3.55, 95% CI 1.94–6.49). Similarly, they were more often exposed to ophthalmological surgery after 1980 (OR: 2.7, 95% CI 1.1–6.5), invasive dental treatment after 1980 (OR 2.63; 95% CI: 1.33–5.19) as well as to regular GP visits (OR 6.33; 95% CI 3.44–11.64) and regular medication (OR 2.3; 95% CI: 1.04–5.10). With respect to a hypothesised iatrogenic transmission, surgical procedures involving lymphoid organs or the central nervous system were not found to be more frequent in sCJD patients than in the controls. Appendectomy, gastro-colonoscopy, tonsillectomy, lumbar punctures, donating blood as well as current cigarette smoking were observed more frequently in the controls than in sCJD patients.

**Table 3:** Medical risk factors – surgery

				**comparison with all control subjects**					
**Risk factor**	**n** **total**	**mean (SD) or %**		**crude estimate**		**age and sex adjusted estimate**		**age, sex and education adjusted estimate**	
		**cases n = 69**	**controls** **n = 224**	**OR (95% CI)**	**p**	**OR (95% CI)**	**p**	**OR (95% CI)**	**p**
surgery, ever	292	94.1	95.1	0.83 (0.25–2.68)	0.751	0.94 (0.28–3.13)	0.921	0.99 (0.29–3.37)	0.984
number of operations	292	3.04 (1.71)	4.33 (2.76)	0.93 (0.81–1.06)	0.260	0.94 (0.82–1.08)	0.377	0.94 (0.82–1.09)	0.410
neurological surgery	289	10.8	7.6	1.47 (0.58–3.71)	0.416	1.51 (0.59–3.82)	0.388	1.24 (0.46–3.33)	0.672
neurological surgery (> 1980)	289	4.6	1.8	2.66 (0.58–12.21)	0.208	2.92 (0.63–13.60)	0.173	2.26 (0.39–13.04)	0.362
ophthalmological surgery	289	15.4	20.5	0.70 (0.33–1.49)	0.357	0.72 (0.33–1.59)	0.417	0.79 (0.35–1.78)	0.574
ophthalmological surgery (> 1980)	289	15.4	8.5	1.96 (0.86–4.46)	0.108	2.23 (0.94–5.28)	0.068	2.67 (1.10–6.49)	0.030
abdominal surgery	288	35.9	50.5	0.55 (0.31–0.98)	0.042	0.56 (0.31–1.01)	0.054	0.53 (0.29–0.98)	0.041
abdominal surgery (> 1980)	288	25.0	18.8	1.44 (0.75–2.79)	0.273	1.49 (0.77–2.88)	0.241	1.44 (0.72–2.85)	0.300
appendectomy	292	22.1	37.5	0.47 (0.25–0.89)	0.020	0.49 (0.26–0.94)	0.032	0.55 (0.28–1.05)	0.071
appendectomy (> 1980)	292	4.4	22.7	0.16 (0.05–0.52)	0.002	0.16 (0.05–0.54)	0.003	0.16 (0.05–0.55)	0.003
gastro- or colonoscopy	285	24.6	44.2	0.41 (0.22–0.78)	0.007	0.43 (0.22–0.81)	0.010	0.45 (0.23–0.87)	0.017
gastro-/colonoscopy (> 1980)	285	9.8	16.5	0.55 (0.22–1.37)	0.201	0.58 (0.23–1.46)	0.250	0.59 (0.23–1.49)	0.260
gynaecological surgery^1^	63	50.0	69.2	0.44 (0.16–1.27)	0.130	0.43 (0.15–1.25)	0.121	0.48 (0.16–1.45)	0.194
gynaecological surgery (> 1980)^1^	63	25.0	33.3	0.67 (0.21–2.08)	0.485	0.57 (0.17–1.94)	0.371	0.77 (0.21–2.80)	0.694
orthopaedic surgery	293	52.2	20.5	4.22 (2.38–7.49)	0.000	4.41 (2.47–7.89)	0.000	4.77 (2.60–8.83)	0.000
orthopaedic surgery (> 1980)	293	44.9	20.1	3.25 (1.82–5.77)	0.000	3.39 (1.89–6.08)	0.000	3.55 (1.94–6.49)	0.000
tonsillectomy	292	35.3	36.6	0.94 (0.54–1.66)	0.844	0.93 (0.53–1.65)	0.806	1.04 (0.58–1.88)	0.889
tonsillectomy (> 1980)	292	2.9	23.7	0.10 (0.02–0.41)	0.002	0.10 (0.02–0.40)	0.001	0.05 (0.01–0.36)	0.003
organ transplantation	292	1.5	0.5	3.33 (0.21–53.93)	0.397	3.36 (0.20–55.81)	0.398	2.69 (0.16–45.67)	0.494

1) female subjects, GP controls only

**Table 4:** Medical risk factors – medication, allergies, invasive procedures, injections, blood transfusions, cigarettes and illegal drugs

				**comparison with all control subjects**					
**risk factor**	**n total**	**mean (SD) or %**		**crude estimate**		**age and sex adjusted estimate**		**age, sex and education adjusted estimate**	
		**cases n = 69**	**controls** **n = 224**	**OR (95% CI)**	**p**	**OR (95% CI)**	**p**	**OR (95% CI)**	**p**
regular GP visits^1^	292	61.8	20.1	6.43 (3.57–11.57)	0.000	6.44 (3.57–11.62)	0.000	6.33 (3.44–11.64)	0.000
regular medication	291	86.6	75.9	2.05 (0.95–4.40)	0.067	2.30 (1.05–5.05)	0.037	2.30 (1.04–5.10)	0.040
hormones, ever	292	14.7	22.3	0.60 (0.29–1.26)	0.177	0.66 (0.29–1.50)	0.319	0.67 (0.28–1.61)	0.366
allergies^2^	135	9.1	14.5	0.59 (0.20–1.73)	0.336	0.66 (0.22–2.05)	0.477	0.59 (0.18–1.95)	0.383
visit to a urologist^3^	171	32.6	39.8	0.73 (0.35–1.51)	0.395	0.75 (0.35–1.58)	0.447	0.79 (0.36–1.74)	0.559
visit to a psychiatrist	292	14.7	12.5	1.21 (0.55–2.63)	0.636	1.16 (0.53–2.58)	0.707	1.33 (0.57–3.12)	0.509
contact lenses	291	1.5	4.5	0.32 (0.04–2.58)	0.287	0.37 (0.05–3.02)	0.355	0.47 (0.06–3.86)	0.482
regular injections	290	16.7	23.2	0.66 (0.32–1.36)	0.259	0.68 (0.33–1.39)	0.285	0.65 (0.31–1.37)	0.256
vaccinations (> 1980)	288	64.1	63.0	1.05 (0.59–1.87)	0.870	1.03 (0.58–1.85)	0.915	1.10 (0.61–2.00)	0.753
acupuncture, ever	290	13.6	21.9	0.56 (0.26–1.22)	0.145	0.57 (0.26–1.26)	0.167	0.60 (0.27–1.34)	0.211
lumbar puncture, ever	289	10.8	25.5	0.35 (0.15–0.82)	0.015	0.34 (0.15–0.80)	0.013	0.32 (0.13–0.79)	0.013
blood transfusions	291	22.4	26.8	0.79 (0.41–1.50)	0.471	0.83 (0.43–1.58)	0.563	0.81 (0.41–1.56)	0.522
blood transfusions (> 1980)	291	6.0	11.6	0.48 (0.16–1.44)	0.191	0.52 (0.17–1.55)	0.240	0.51 (0.17–1.54)	0.231
blood donation	290	16.7	47.8	0.22 (0.11–0.44)	0.000	0.16 (0.07–0.33)	0.000	0.16 (0.07–0.34)	0.000
invasive dental treatment (> 1980)	290	78.8	62.5	2.23 (1.16–4.27)	0.016	2.25 (1.17–4.32)	0.015	2.63 (1.33–5.19)	0.006
tattoo's	290	1.5	1.8	0.85 (0.09–7.70)	0.882	0.91 (0.10–8.34)	0.933	0.87 (0.09–8.14)	0.906
piercings/earrings	290	13.4	19.6	0.63 (0.29–1.38)	0.251	0.73 (0.30–1.78)	0.483	0.73 (0.29–1.83)	0.503
current cigarette smoker	284	13.3	29.0	0.38 (0.17–0.84)	0.016	0.34 (0.15–0.76)	0.009	0.34 (0.15–0.78)	0.010
illegal drugs	289	1.5	4.5	0.33 (0.04–2.66)	0.301	0.32 (0.04–2.53)	0.278	0.37 (0.05–3.04)	0.355

1) other than for CJD 2) GP controls only 3) male subjects only

#### Dietary factors

In this analysis, among the 20 food items analysed, only the age-, sex- and education-adjusted estimate of kidney consumption showed a significant association with sCJD (OR: 1.96, 95% CI 1.04–3.68), but this difference was rather weak. Results are shown in table 5.

**Table 5:** Dietary risk factors – food habits: meat, innards and dairy products (> 1980)

				**comparison with all control subjects**					
**Risk factor**	**n** **total**	**mean (SD) or %**		**crude estimate**		**age and sex adjusted estimate**		**age, sex and education adjusted estimate**	
		**cases n = 69**	**controls n = 224**	**OR (95% CI)**	**p**	**OR (95% CI)**	**p**	**OR (95% CI)**	**p**
vegetarian for > 1 year	290	3.0	5.4	0.55 (0.12–2.53)	0.445	0.62 (0.13–2.93)	0.550	0.81 (0.17–3.95)	0.798
cow's milk (regularly/daily)	288	87.7	87.9	0.98 (0.43–2.28)	0.965	0.98 (0.42–2.30)	0.962	1.00 (0.42–2.39)	0.997
cheese (> 1/week)	288	95.4	96.0	0.87 (0.23–3.31)	0.837	0.84 (0.22–3.22)	0.800	0.68 (0.17–2.71)	0.588
beef (> 1/week)	287	50.8	54.5	0.86 (0.49–1.50)	0.595	0.84 (0.48–1.47)	0.549	0.88 (0.50–1.55)	0.650
veal (> 1/week)	286	40.6	30.6	1.55 (0.87–2.75)	0.135	1.51 (0.84–2.69)	0.167	1.55 (0.86–2.80)	0.142
pork (> 1/week)	287	63.1	62.6	1.02 (0.58–1.81)	0.946	0.99 (0.55–1.76)	0.960	0.93 (0.52–1.68)	0.811
lamb, ever	287	81.5	86.0	0.72 (0.34–1.49)	0.373	0.63 (0.29–1.34)	0.229	0.74 (0.34–1.64)	0.463
game/venison, ever	285	87.5	81.5	1.59 (0.71–3.60)	0.262	1.45 (0.63–3.35)	0.384	1.48 (0.64–3.45)	0.362
chicken (> 1/week)	285	63.5	57.7	1.28 (0.73–2.33)	0.407	1.28 (0.71–2.28)	0.411	1.23 (0.68–2.21)	0.495
sausages (> 1/week)	283	54.1	42.8	1.58 (0.89–2.78)	0.118	1.51 (0.85–2.69)	0.161	1.55 (0.86–2.78)	0.147
hamburgers at restaurants, ever	286	37.5	24.8	1.82 (1.01–3.29)	0.047	1.76 (0.95–3.24)	0.070	1.74 (0.93–3.26)	0.086
innards, ever	287	56.9	50.5	1.30 (0.74–2.27)	0.359	1.27 (0.72–2.23)	0.404	1.32 (0.75–2.35)	0.337
liver, ever	287	78.5	73.9	1.29 (0.66–2.50)	0.454	1.28 (0.66–2.49)	0.469	1.23 (0.62–2.47)	0.552
kidneys, ever	287	33.9	22.5	1.76 (0.96–3.22)	0.066	1.68 (0.91–3.09)	0.094	1.96 (1.04–3.68)	0.037
liver sausage/paté, ever	285	66.7	62.6	1.19 (0.66–2.15)	0.556	1.16 (0.64–2.11)	0.620	1.16 (0.64–2.13)	0.626
blood sausage/black pudding, ever	285	57.1	54.1	1.13 (0.64–1.99)	0.664	1.11 (0.63–1.95)	0.729	1.12 (0.63–2.00)	0.696
tartare/carpaccio, ever	286	31.3	36.0	0.81 (0.44–1.46)	0.480	0.75 (0.41–1.38)	0.362	0.84 (0.45–1.56)	0.585
brain, ever	287	4.6	9.9	0.44 (0.13–1.52)	0.194	0.42 (0.12–1.47)	0.177	0.51 (0.14–1.79)	0.290
eyes, ever	287	1.5	0.5	3.45 (0.21–55.99)	0.383	3.00 (0.18–49.06)	0.441	5.59 (0.33–96.89)	0.235
cat-food, ever	290	0.0	1.3	--	--	--	--	--	--

#### Subgroup analysis

Results for most of the factors were similar when analysed for both control groups separately. Factors with significant differences between the cases and the composite control groups also displayed significant differences when cases were compared to the two control groups separately, as was the case for number of school years for example, while on the other hand most factors without such differences in the combined control-group analysis did not display significant differences when the two control groups were analysed separately.

However, there were a few exceptions to this. In the adjusted analyses, sporadic CJD patients compared to just the GP controls were more likely to have consumed kidney meat (OR: 3.17; 95% CI 1.27–7.94), hamburgers at restaurants (OR: 3.26; 95% CI 1. 30–8.21), or ever eaten game meat or venison (OR: 2.68; 95% CI 1.03–7.02). Sporadic CJD patients were less likely than the GP controls to have dementia in the family history (OR: 0.271; 95% CI 0.07–0.85), to have had surgical interventions (OR: 0.811; 95% CI 0.68–0.96) and gastro- or colonoscopy after 1980 (OR: 0.178; 95% CI 0.06–0.49), to have received blood transfusions (OR: 0.222; 95% CI 0.07–0.72), and to own a cat (OR: 0.435; 95% CI 0.20–0.92).

When compared to just the telephone controls, in the adjusted analyses, sCJD patients were more likely to have undergone invasive dental treatment (OR: 3.11; 95% CI 1.55–6.25), ophthalmologic surgery performed after 1980 (OR: 18.31; 95% CI 3.65–91.97), have taken medicaments regularly (OR: 2.84; 95% CI 1.27–6.32) and to consume sausages more than once a week (OR: 2.22, 95% CI 1.18–4.18), and less likely to have travelled to the UK (OR: 0.256; 95% CI 0.09–0.76) or to tropical countries (OR: 0.405; 95% CI 0.18–0.93), to have undergone appendectomy (OR: 0.476; 95% CI 0.24–0.94) and lumbar puncture (OR: 0.261; 95% CI 0.10–0.65), to have ever smoked (OR: 0.269; 95% CI 0.12–0.62), to own a pet rodent (OR: 0.220; 95% CI 0.11–0.46).

## Discussion

This study evaluated a wide range of possible risk factors as risk factors for sCJD-cases observed in Switzerland between 2001 and 2004. If some of the positively associated risk factors were truly causal and would have become more frequent in recent years and decades, then they might have contributed to the increase in sCJD-cases in Switzerland. Although some analysed factors were significantly more frequent in the group of sCJD patients, the results of this case-control study have not produced unequivocal evidence for specific environmental or iatrogenic risk factors for sCJD, and thus could not reveal a specific explanation for the increased incidence. In interpreting the results, one has to bear in mind that with a significance level set at 5%, on average one in twenty results will be significant, by chance alone.

Starting with the hypothesis of a zoonotic cause similar to the development of vCJD by consumption of BSE-contaminated material, the present study does not demonstrate a clearly increased risk for sCJD with respect to dietary habits. Although previous studies revealed that consumption of several meat products was increased in sCJD cases compared to controls,[26,27] no such clear differences for any of the twenty diet-related exposures were found in our as well as in earlier studies. [28-30] Consistent with previous studies, no significant differences between sCJD patients and controls was found with respect to occupational exposures, including work in animal farming or in the meat industry in the present study. One exception was work in an animal laboratory. [27-31] Some findings of earlier studies which revealed higher frequencies of sCJD among butchers,[32] among those exposed to leather products, to fertiliser consisting of hoofs and horns,[27] and to farm-stays for any length of time,[31] could not be reproduced in this study. Contrary to the hypothesis of a zoonotic cause, earlier studies did not link pet animals possession to increased risk. [27-30] Patients in this study even less often owned cats or pet rodents. Travelling abroad was significantly more frequent in sCJD patients. Controls, however, travelled more frequently to the United Kingdom where risk for vCJD was elevated and to tropical destinations where some infectious diseases are more prevalent than in Switzerland. No differences in age, sex, place of birth and living or family history of dementia were found. These results are consistent with data from previous studies.[26,28,29] In contrast to earlier studies, sCJD-patients in our study had completed less years of education.[26,28] Only 45% of the cases had ten or more school years, whereas this figure was at 71% among the controls. These findings might be due to recall bias which can occur when interviewing proxy persons. However, another neurodegenerative disease, Alzheimer's disease has been shown to be observed more frequently in persons with lower levels of education.[33]

Concerning the hypothesis of an iatrogenic cause, sCJD cases in this study significantly more often had undergone orthopaedic and ophthalmologic surgery, invasive dental treatment as well as regular medical treatment. In previous studies, physical injuries and stressful life events such as surgical procedures,[23,28,30,31,34,35] and work in a medical profession[32] have been found to constitute a risk factor for sCJD. In particular, head surgery and trauma to other body parts were identified.[36] In contrast to the present study, however, orthopaedic surgery and invasive dental treatments per se were not associated with an elevated risk for sCJD before.[27,28,31,36] One could speculate that the use of surgical instruments as well as a potentially higher rate of blood transfusions in orthopaedic surgery may explain these findings. Receiving blood transfusions has been demonstrated to be a potential route of vCJD transmission.[20] The development of vCJD involves a peripheral route of prion transmission to the CNS. In sCJD, however, the disease most likely starts in the brain, even though recently, prions have been detected in peripheral organs such as the spleen and skeletal muscles of sCJD patients.[37] Blood transfusions, however, were not associated with sCJD in the present study. Interestingly, blood donation was less frequently observed in sCJD patients than in controls. Correspondingly, neither receiving blood transfusions nor blood donation was identified as a risk factor for sCJD in previous studies.[23,26,38,39] Cigarette smoking was more frequent in controls (29%) than in sCJD patients (13%) or in the general population, a finding which points to some bias in the selection of controls. A previous analysis did not find any association with smoking.[26]

Recently, the heightened incidence of sCJD in Switzerland was found to be associated with a shift in clinicopathological profiles, in that sporadic CJD patients from the cohort with elevated sporadic CJD incidence presented with a higher frequency of rare sporadic CJD-subtypes (MV2, VV2). Patients of these subtypes were significantly older and showed a skewed male/female ratio when compared to patients of identical sporadic CJD-types or to patients from the 1996–2000 cohort.[22]

The third hypothesis to explain the increase in annual mortality rates from sCJD in Switzerland between 2001 and 2004 is a better case ascertainment. Over recent years in Europe, as a general tendency incidences have been rising, however not to such extent as in Switzerland. Given that our results do not support strong evidence for the hypotheses of a zoonotic or iatrogenic cause, ascertainment bias due to a heightened perception and awareness of the disease in physicians must be regarded as the most likely cause for the observed increase. One factor that might be jointly responsible for this increase might be altered reporting requirements in 1999. Since that year all suspected cases in Switzerland had to be reported. In this respect, also the role of chance must be considered, as it is possible that the observed increase of Swiss sCJD-deaths was due to random fluctuation. The rise in annual mortality rate from the years before 2000 to the period 2001–2004, however, was statistically significant, and therefore, chance must be considered a less likely explanation. In the most recent years, the observed incidence in sCJD deaths (2005: 10; 2006: 13, 2007: 15) dropped to levels just slightly above those before 2000. When recent incidence data until 2007 are included, however, the rise in the annual mortality rate after 2001 was still significant. The sudden increase in 2001 and the slow decrease afterwards are well in line with the media coverage of the CJD topic in the respective years.

### Strengths and limitations

This study was initiated to clarify whether cases of sCJD from 2001 to 2004 were associated with a specific environmental risk factor in Switzerland. A well defined group of patients was examined. The 69 sCJD cases consisted of all but three known cases in Switzerland during the study period. The participation rate of relatives and friends of cases was high, and for cases and controls there were only few missing data points in the data sets. The study used a standardised questionnaire with a wide range of potential risk factors reported in previous studies. However a subtype analysis in correlation with risk factors was not performed in this analysis because of the small number of cases.

Due to Switzerland's small population size, the rarity of the disease, and the fact that sCJD invariably leads to death within less than 2 years, the number of cases in the study was relatively small. Other case-control studies conducted in Europe were somewhat larger. However, of these studies, just four recent studies also included data of cases from the year 2001 or later,[32,40-42] and none was performed within Switzerland. Consequently, they could not contribute to an explanation for the increased incidence of reported sCJD deaths in Switzerland. Two different control groups were chosen to limit selection bias. The interviews of controls by telephone yield potential for self-selection bias in that ownership of a fixed telephone line or willingness to participate might be unevenly distributed among the general population. On the other hand, the control-group of patients from GP offices were matched more closely but certain illnesses and other factors may be overrepresented in this group. The interviewed control persons answered the questions directly – either by a face-to-face interview or by telephone, whereas for the cases a close relative or friend of the patient answered the questions as a proxy. Furthermore, interviewed persons were conscious about the aims of the study, all of which could have resulted in the introduction of recall bias. Interviewing a proxy person for sCJD cases could imply that responses were less accurate, with fewer positive responses with respect to potential risk factors, potentially masking differences between the cases and controls. Conversely, a face-to-face interview could have resulted in more accurate data than a telephone interview. For instance, in the subgroup analysis comparing ophthalmologic surgery after 1980 in patients and telephone controls, the finding of an odds ratio of 18.31 with a very wide 95% C.I. of 3.65–91.97 has to be interpreted with care. Only 2 of 155 telephone controls (1.3%) recalled having undergone ophthalmologic surgery after 1980, while in the face-to-face interviews this proportion was 15.4% among proxy persons for patients and 24.6% among the GP controls. In the present study, we renounced interviewing proxy persons for controls, since we hypothesised that proxy-persons for cases would be more disposed to answer such questions correctly than proxy-persons for controls would do, and that such an effort would not have excluded recall bias.

### Unanswered questions, future research

To identify environmental or other causes of sCJD, further research is necessary. The questionnaire of our study has been used in other CJD case-control studies,[23,24] which will allow to combine our results with those from other studies. As a potential risk factor for prion infection, ear, nose and throat (ENT) – surgery was not included as an item in this study. Other than recall bias, we could not find an explanation, why some of the examined factors, such as the number of school years differed between sCJD cases and controls.

In this study, no genetic markers were analysed. For practical and ethical reasons, this information was only available for patients and not for controls. However, such information could have given additional clues, in that the development of sporadic CJD due to environmental factors might be linked to some so far unknown genetic factors. More recent research indicates that there are different subtypes of sCJD. Patients have a wide range of genetic backgrounds, which results in different clinical and histopathological presentations and diverse PrPSc distribution patterns in the brain.[43]

## Conclusion

This study did not reveal a specific explanation for the increased annual death rates for sCJD observed in Switzerland between 2001 and 2004 with respect to external – and potentially avoidable – risk factors. Although some factors were significantly more frequent in the group of sCJD cases, the results have to be interpreted with caution due to fact of having tested multiple hypotheses and due to the possibility of bias in the selection of controls and of recall bias in association with the potentially long incubation period as seen in other prion diseases. The most plausible reason for the observed increase in 2001 in Swiss sCJD cases is an improved case ascertainment. The improved reporting of cases was in temporal correlation with the rise in variant CJD cases in the United Kingdom and the resulting high media coverage. Therefore, underreporting of cases might well have occurred before the year 2001, and the "real" yearly incidence of sCJD might not be lower than, but rather above 2 per million inhabitants.

## Competing interests

The authors declare that they have no competing interests.

## Authors' contributions

JR collected data of control subjects and participated in the writing of the manuscript. KS participated in the design of the study, collected data of case subjects and participated in the writing of the manuscript. LAdesigned the study and participated in the interpretation of data. TB participated in the design of the study and collected data of case subjects. MZ participated in the design of the study, in data analysis and interpretation and revised the manuscript critically. AA participated in the collection of data of case subjects and revised the manuscript critically. MG participated in the design of the study, in the collection of data of case subjects and revised the manuscript critically. KH participated in the collection of data of control subjects and in the writing of the manuscript, and revised the manuscript critically. TE collected data of control subjects, participated in data analysis and interpretation, and in the writing of the manuscript.

## Pre-publication history

The pre-publication history for this paper can be accessed here:


